# The impact of disinvestment on alcohol and drug treatment delivery and outcomes: a systematic review

**DOI:** 10.1186/s12889-021-12219-0

**Published:** 2021-11-22

**Authors:** Suzie Roscoe, Jennifer Boyd, Penny Buykx, Lucy Gavens, Robert Pryce, Petra Meier

**Affiliations:** 1 School of Health and Related Research, University of Sheffield, Regent Court, 30 Regent Street, Sheffield, S1 4DA UK; 2 School of Humanities and Social Science, University of Newcastle, University Drive, Callaghan, NSW 2308 Australia; 3 MRC/CSO Social and Public Health Sciences Unit, University of Glasgow, Berkeley Square, 99 Berkeley Street, Glasgow, G3 7HR UK

## Abstract

**Background:**

In the context of substantial financial disinvestment from alcohol and drug treatment services in England, our aim was to review the existing evidence of how such disinvestments have impacted service delivery, uptake, outcomes and broader health and social implications.

**Methods:**

We conducted a systematic review of quantitative and qualitative evidence (PROSPERO CRD42020187295), searching bibliographic databases and grey literature. Given that an initial scoping search highlighted a scarcity of evidence specific to substance use treatment, evidence of disinvestment from publicly funded sexual health and smoking cessation services was also included. Data on disinvestment, political contexts and impacts were extracted, analysed, and synthesized thematically.

**Results:**

We found 20 eligible papers varying in design and quality including 10 related to alcohol and drugs services, and 10 to broader public health services. The literature provides evidence of sustained disinvestment from alcohol and drug treatment in several countries and a concurrent decline in the quantity and quality of treatment provision, but there was a lack of methodologically rigorous studies investigating the impact of disinvestment.

**Conclusions:**

This review identified a paucity of scientific evidence quantifying the impacts of disinvestment on alcohol and drug treatment service delivery and outcomes. As the global economy faces new challenges, a stronger evidence base would enable informed policy decisions that consider the likely public health impacts of continued disinvestment.

**Supplementary Information:**

The online version contains supplementary material available at 10.1186/s12889-021-12219-0.

## Background

Addressing the burden of alcohol and drug harm through the provision of treatment is a global priority [1]. Treatment for substance use disorders reduces health and social harms from alcohol and drugs, providing a good return on investment [2–9]. Many countries which publicly fund alcohol and drug services have been faced with large reductions in spending power, resulting in disinvestment from alcohol and drug treatment [10–13].

In England, increased investment in treatment in the early twenty-first century, was associated with improved treatment access, reduced waiting times, improved service quality and a reduction in related harm [14–16]. Since 2012, there have been substantial changes to how drug and alcohol treatment in England is funded. The Health and Social Care Act 2012 transferred public health responsibilities, including the budget for alcohol and drug treatment, from the National Health Service to Local Authorities (local government organisations; N*.* 152 in England) [17]. At the same time a ring-fence protecting the alcohol and drug budget was removed, although protection for the total public health budget remained [18]. This transfer coincided with a period of public sector austerity in the wake of the global recession, with significant budget reductions for local government across a wide range of responsibilities [19, 20].

There have been widely reported changes to the investment in alcohol and drug treatment since 2014/15, with overall reductions in the amount local governments are investing in these services [21, 22]. Concurrently, trends in routine monitoring data show declines in treatment outcomes and increases in alcohol and drug related deaths and alcohol-related hospital admissions, with substantial variation across the country [23–25].

Whilst there is a strong evidence base for the effectiveness, and return on investment, of alcohol and drug treatment, the impact of recent disinvestment from these services remains unclear. Therefore, it is of policy interest and timely to synthesise available literature. An initial scoping search focused on alcohol and drug treatment revealed a paucity of evidence and therefore this review also considers what can be learnt from literature about disinvestments from similar local authority public health services, namely sexual health and smoking cessation services, which have also faced cuts [26, 27].

This review addressed the following questions:

i. What is the impact of disinvestment from publicly funded alcohol and drug treatment for adults in England?
ii. What is the impact of disinvestment from publicly funded alcohol and drug treatment for adults in other Organisation for Economic Co-operation and Development (OECD) countries?
iii. What can we learn from the impact of disinvestment from other publicly funded public health programmes, specifically smoking cessation and sexual health programmes, in England and other OECD countries?

## Methods

### Protocol, registration and search strategy

Following an initial scoping search, a pre-specified protocol was developed and registered on the International Prospective Register of Systematic Reviews (PROSPERO, CRD42020187295). We undertook a systematic search of the following bibliographic databases in July 2020: EMBASE (1980 to June 2020), MEDLINE (1946 to June 2020) and CINAHL (1981 to June 2020). An extensive list of search terms was used against each of the above research questions. To identify additional relevant, including grey, literature backward searching of citations was completed and www.evidence.nhs.uk and Google Scholar were searched using simplified search terms, for example, “cuts to alcohol and drug treatment”.

### Inclusion criteria

Journal publications and grey literature pertaining to the review questions and search strategy were included. This included primary and secondary quantitative and qualitative research examining the impact of disinvestment from the following publicly funded services: alcohol and drug, sexual health and stop smoking services. Relevant journal-published opinion pieces and grey literature from credible sources were also included. Any described or measured impacts related to disinvestment were included - for example, changes to the way services were commissioned or provided, treatment access and completion rates, and broader health and social implications. Sexual health and smoking cessation literature was included to enable learning to be drawn from comparable, large investment services that may have experienced budget cuts [28]. Additional inclusion criteria were literature that was: published in English; focused on OECD countries; services publicly funded for example, by a government body or a national health organisation.

### Data extraction and analysis

Titles and abstracts of citations were screened within the bibliographic databases and those meeting the eligibility criteria were imported to EndNote, and duplicates were removed. Full texts were reviewed to dictate inclusion or exclusion before a data extraction table was compiled. Each paper was quality assessed using the most appropriate available tool for the reported study design via the Critical Appraisal Skills Programme (CASP) and the Joanna Briggs Institute (JBI) [29, 30]. The grey literature were appraised via the Authority Accuracy Coverage Objectivity Date Significance (AACODS) checklist [31]. The selection of the most appropriate critical appraisal tool was not always straightforward but is detailed within the supplementary information. For example, the Freudenberg et al. paper [32] was reviewed using the CASP systematic review checklist as the paper is a peer-reviewed synthesis of relevant literature. However, it does not follow a systematic review design and therefore it is unclear whether all relevant papers were included, or if included papers were assessed for quality. Furthermore, the diversity of included publication types means that some were unlikely to have been written with quality appraisal in mind. For example, within the grey literature, the limitations and bias of the content covered (or the research undertaken) were not always explicit, which impacted on the ability to assess the overall accuracy of the papers.

The papers were then analysed thematically, adopting Braun and Clarke’s approach to qualitative data [33], and synthesised narratively, using the Synthesis Without Meta-analysis protocol [34]. SR led the search, data extraction and analysis and JB reviewed all papers to confirm eligibility, and completed thematic analysis of half of the papers, prior to discussion and agreement of final themes. JB also independently quality appraised a random sample of 25% of included papers. Given the heterogeneity of the papers and that no study attempted to quantify the primary question, no weighting of results was applied according to, for example, whether claims are substantiated by empirical findings. Instead, an inductive thematic approach was used to explore conceptual similarities across heterogeneous literature to provide an overview of the politico-economic context of any disinvestments, related changes to provision and outcomes. The extraction tables (Tables 1 and 2) provide details of the publication and / or study type.

**Table 1:** Extraction table of literature specific to examining the impact of disinvestment from alcohol and drug treatment

Author and year published	Paper title	Peer reviewed	Population	Country setting	Sample size	Focus of paper	Method(s)	Publication type	Findings
Adfam, 2017 [15]	Commissioning impact on drug treatment	No	Stakeholders - providers, commissioners, Police and Crime Commissioner, Directors of Public Health, National probation service	England	23	Alcohol and drug treatment	Mixed methods: semi-structured interviews and secondary data analysis via convenience and snowballing sampling	Charitable organisation primary research report	Disinvestment thus far has not resulted in diminished quality or safety of the provision of alcohol and drug treatment services. Further service development is required to respond to need. Concerns about future cuts.
Advisory Council on the Misuse of Drugs, 2017 [38]	State of the Sector: Beyond tipping point	No	149 commissioning teams of drug treatment	England	106	Drug treatment	Mixed methods: literature review, secondary data analysis, survey, and statements from professional bodies	Statutory advisory non-departmental public body primary research report	Disinvestment is the biggest threat to drug treatment and achievement of recovery outcomes. Concerns regarding service quality and effectiveness, disconnection from other health services and impact of re-tendering.
Alcohol concern, 2014 [45]	A measure of Change: an evaluation of the impact of the public health transfer to local authorities on alcohol	No	England’s alcohol treatment providers and local authorities and Clinical Commissioning Groups	England	75	Alcohol treatment	Quantitative: two cross-sectional surveys	Charitable organisation primary research report	Majority of alcohol treatment services had maintained or increased funding. Concerns that areas of high harm least likely to increase funding. Treatment providers less optimistic than local authorities about funding. Funding for alcohol treatment is insufficient for its priority focus.
Alcohol concern, 2018 [39]	The hardest hit: addressing the crisis in alcohol treatment services	No	Mailing list of Alcohol Concern’s consultancy and training and “friends.” Includes range of professionals and service users	England	154 Surveys and 40 interviews	Alcohol treatment	Mixed methods: secondary data analysis, cross-sectional survey and telephone interviews	Charitable organisation primary research report	Reported insufficient funding of alcohol treatment and reduced workforce. Majority of stakeholders reported re-tendering within last three years. Mixed views regarding alcohol and drug service integration. Concerns regarding insufficient support for those with complex needs and older drinkers.
Blenheim, 2018 [40]	Failure by design and disinvestment	No	Alcohol and drug treatment provision in criminal justice settings	England and Wales	N/A	Alcohol and drug treatment	Opinion / Review of existing research	Charitable organisation research report	Concerns about disinvestment and its relationship to a reduction in the quality of support during transition from custody to community services for people dependent on drugs.
Cook (Harm Reduction International), 2017 [41]	Harm reduction investment in the European Union current spending, challenges and successes	No	Harm reduction leads from 18 countries	Europe	18 EU member states	Drug treatment	Quantitative: cross-sectional survey and secondary data analysis	Non-Government Organisation research report	Future sustainability of harm reduction varies from fairly certain, to extremely insecure. Public sector austerity, reductions in international donors and poor political support were perceived as factors contributing to the poor funding of harm reductions.
Drink and drug news, 2018 [44]	On a knife edge	No	Drug treatment population	UK	N/A	Drug treatment	Journalism	Magazine article	Concerns that disinvestment has contributed to a reduced focus on, and delivery of, harm reduction.
Hayes, 2018 [43]	At the heart of the matter	No	Alcohol and drug treatment population	UK	N/A	Alcohol and drug treatment	Opinion piece	Magazine feature	Concerns regarding disinvested and reduced treatment offer despite insufficient reach of alcohol services, increasing drug-related deaths, fragmentation from health services and increases in drug-related crime.
Mohammadi, 2014 [42]	Addiction services in England: in need of an intervention	No	Stakeholders within alcohol and drug treatment sector, including clinicians, consultants and commissioners	England	Quotes from six sector stakeholders	Alcohol and drug services	Editorial, including quotes from stakeholders	Journal opinion piece	Exploration of changes in way services are commissioned. Changes from NHS to non-NHS providers and mixed views about the effects in terms of specialism and appropriateness for treatment population.

**Table 2:** Extraction table of literature examining the impact of disinvestment from public health services

Author and year published	Paper title	Peer reviewed	Population	Country setting	Sample size	Focus of paper	Method(s)	Publication type	Findings
Anderson et al., 2017 [35]	Political priorities and public health services in English local authorities: the case of tobacco control and smoking cessation services	Yes	152 Tobacco control leads from each upper tier authority	England	116 in 2014; 124 in 2015 and 129 in 2016	Smoking cessation services in England	Quantitative: cross-sectional survey. Longitudinal comparing 87 local authorities	Journal study	Political support for tobacco control mitigates the risk of cuts to smoking cessation budgets.
British Medical Association, 2018 [46]	Feeling the squeeze. The local impact of cuts to public health budgets in England	No	Public Health Professionals	England	N/A	Public health services	Quantitative: Secondary data analysis	Professional body research report	Changes in public health spending do not reflect the needs of local populations. Disinvestment leading to variation in quality and quantity of service provision.
Chang, 2010 [37]	Quit smoking advice from health professionals in Taiwan: The role of funding policy and smoker socioeconomic status	Yes	Participants of the Taiwan Adult Tobacco Survey	Japan	16,688 in 2004, 16,749 in 2005, 16,922 in 2006 and 16,588 in 2007	Smoking cessation services in Japan	Quantitative: secondary data analysis	Journal study	Quit prevalence increases were associated with increases in funding. Quit prevalence reduced, but not significantly, following disinvestment.
Davies et al. (Quality Watch), 2016 [47]	Focus on: Public Health and prevention	No	120 Directors of Public Health, service providers and advocacy organisations	England	37 for survey and 11 interviews	Public health services	Mixed methods: secondary data analysis, cross-sectional survey and interviews	Health think tank research report	6/10 public health indicators deteriorated between 2009 and 15, including alcohol-related hospital admissions but completion of substance use treatment improved. Positive views regarding local government procurement processes but concerns regarding effect of financial pressures on service accessibility and effectiveness.
Daube, 2012 [10]	A bleak outlook for public health?	No	Government funded public health programmes	Australia	N/A	Public health services	Editorial	Journal editorial	Concerns regarding the impact of public sector austerity on public health services, on de-prioritisation of public health, loss of specialist staff, and the withdrawal of specialist services to reduce inequalities. Concerns government legislative changes are at odds with public health ambitions.
Freudenberg et al., 2006 [32]	The impact of New York City’s 1975 Fiscal Crisis on the tuberculosis, HIV, and homicide syndemic	Yes	New York City’s population	US	N/A	Drug treatment and other public services	Secondary data analysis and literature review	Journal study	Estimated that $10 billion cuts to public services, including public health, resulted in $50 billion costs in controlling the TB, HIV and homicide endemics**.**
Iacobucci, 2014 [48]	Raiding the public health budget	No	152 Upper Tier local authorities	England	143	Public health services	Editorial - Freedom of information request analysis	Journal opinion piece	Concerns regarding increasing use of public health grant to support broader local authority services and variation in commissioning across the country.
Iacobucci, 2016 [49]	Public health - the frontline cuts begin	No	152 Upper Tier local authorities	England	132	Public health services	Editorial - Freedom of information request analysis	Journal opinion piece	Decrease in public health grant and concurrent cuts to frontline public health services.
McFarlane and Meier, 1993 [36]	Restructuring Federalism: the impact of Reagan Policies on the Family Planning Program	Yes	Population to benefit from family planning programmes	U.S.	N/A	Family planning services	Secondary data analysis and literature review	Journal study	Disinvestment from family planning services concurrent to a reduction in people supported and increased variation in services within more deprived groups.
Robertson et al., 2017 [50]	Understanding NHS financial pressures (from p26)	No	Population to benefit from GUM services	England	99 stakeholders from NHS	Sexual health services (and other NHS funded services)	Qualitative: semi-structured interviews	Charitable organisation research report	Continued financial pressures on services and for sexual health services, evidence of reduced accessibility and quality of provision. Increasing gap between demand and availability. Commissioners working to identify ways to maintain services.
White, 2016 [51]	Sexual health services: divided and unprotected	No	152 Upper Tier local authorities	England	150/152 local authorities	Sexual health services	Editorial - Freedom of information request analysis	Journal opinion piece	Large variation in local authority prioritisation of sexual health, and related investment in services. Evidence of cuts / planned cuts to sexual health services despite need.

## Results

### PRISMA diagram

**Fig. 1:**
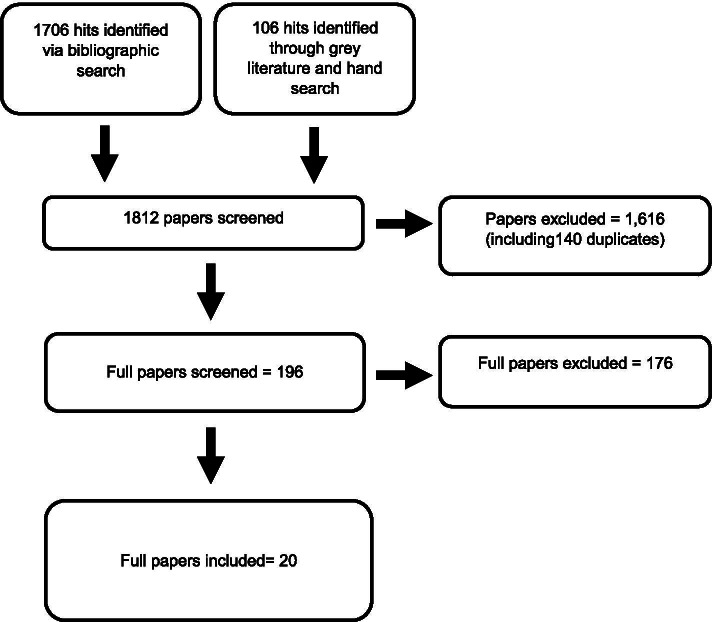
PRISMA diagram

Figure 1 shows the flow of articles through the review process. Database and grey literature searches returned 1812 records; of which 196 underwent full text screening. Twenty papers were included in the review.

### Settings and quality of papers

#### Study characteristics and quality

Of the 20 eligible papers, 13 were research papers, five journal editorials and two substance misuse professional magazine articles. Ten papers related to alcohol and drugs services, three to sexual health services, two to smoking cessation services and five to public health services more generally. Table 1 provides data extracted from the papers explicitly focused on disinvestment from alcohol and drug treatment services and Table 2 shows data from the wider papers. Four of the research papers were peer reviewed: one English study analysing results from a survey of local government tobacco leads regarding smoking cessation services [35], two US studies exploring data and literature on specific public health policy and funding [32, 36], and one Japanese study analysing secondary survey and routine finance data examining the relationship between (dis)investment and smoking cessation advice [37]. Six of the remaining research papers focused on substance use [15, 38–41] and were UK (*n =* 5) and multi-country European (*n =* 1) based. One of the five journal editorials [42] and both magazine articles [43, 44] were substance use specific, the remainder focussed on broader public health services. The majority of these were from the UK (UK *n =* 6, Australia *n* = 1). The overall quality of included papers according to quality appraisal was modest. However, due to the limited number of relevant papers identified, no papers were excluded on the basis of low quality. No studies that attempted to examine a quantifiable or causal relationship between disinvestment from substance use services and treatment delivery or outcomes were identified. Instead, the studies tend to focus on changes in treatment provision and related health outcomes, concurrent or subsequent to disinvestment.

### Thematic synthesis

Three major themes were identified: i) diminished quantity and quality of services; ii) changed commissioning systems and practices; and iii) health, social and broader implications. We present findings relating to each of these themes in turn.

### Diminished quantity and quality of services

The literature offers insights to how services offered have changed in the wake of disinvestment, often relating a decline in the availability of treatment and a deterioration in the quality of support offered [10, 15, 32, 38, 40, 41, 43, 45–47].

Initial cuts to alcohol and drug treatment services were purported to have provided opportunities to find efficiencies and drive service reform [15], and to focus on a greater return on investment [47]. However, continued cuts were described as detrimental to service availability and quality [15, 38, 40, 41]. Organisational research details stakeholder concern that the funding available for alcohol and drug treatment has become increasingly insufficient [15, 39, 47], and is mismatched to the vision for “gold-standard” treatment services in recent clinical guidelines [15, 21, 43].

As budget cuts continued, specific interventions and treatment modalities including harm reduction [41, 44] and residential rehabilitation [39] were regarded as under particular threat. Mixed methods studies targeting treatment sector stakeholders revealed concerns about increasing caseloads, fewer appointments, the replacement of one-to-one work with group sessions, reduced harm reduction and less outreach support [15, 38, 39, 41, 45–47]. Similar changes have been experienced in smoking cessation and sexual health services following disinvestment, referencing a propensity to focus on acute care when budgets are tight [49–51]. This latter concern has also been raised specifically in relation to the alcohol and drug sector, suggesting that services were having to revert to focussing solely on maintenance prescribing [43].

In addition to changes in the treatment offered, there were reports of a reduction in the number of people accessing [15, 32, 40] and successfully completing alcohol and drug treatment [47]. This echoes experiences following disinvestment from sexual health services in the UK [50, 51], from drug treatment in the US [32], and from smoking cessation support in Japan [37]. In Japan, additional effects were seen following disinvestment, including reduced stakeholder engagement and fewer smoking cessation media campaigns [37].

Substantial changes in the alcohol and drug treatment sector during a period of disinvestment were purported to have contributed to an increasingly deskilled and disenfranchised workforce [15, 32, 38]. This included examples of an overreliance on volunteers who had replaced paid staff [15, 38], a loss of specialist positions (such as addiction psychiatrists for more generic clinician roles) [42], and a reduction in the amount of training for the sector’s workforce [15, 37, 38, 42].

### Changed commissioning systems and practices

The processes and systems that exist to commission public health services also appeared to have changed substantially. Subsequent to the transfer of public health responsibilities to local authorities, the stretch on financial resources affected commissioning systems and practices [15, 32, 35, 39, 41, 46]. This included resulting changed responsibilities, procurement activity and fragmentation, with large variation across local authorities.

A growing number of local government areas in England are reported to have integrated various public health services into combined contracts, including the merger of community alcohol and community drug services [15, 39, 48]. Limited attention has been given to the rationale for this move but budget efficiencies are cited in some cases [36, 40], and these mergers have been criticised for reducing service effectiveness [36, 49]. Alcohol and drug treatment sector stakeholders raised concerns that integration can weaken evidence-based practice and that the merger of alcohol and drug services might result in a disproportionate, or diluted, provision for the alcohol treatment population [39].

Whilst it is unclear as to whether the number of retendering exercises has increased, the frequency and process of retendering of alcohol and drug services has been described as hindering outcomes and detracting from frontline delivery of services for a period of up to 18 months [15, 38–40, 42, 47, 48]. There has also been a rise in the use of payment by results, aligning all or partial contract payment to the achievement of specific goals, such as abstinence. Though recognised as an option for achieving a greater return on investment, such payment schedules are perceived as side-lining a client group for whom abstinence is not a goal [38, 42].

Disinvestment has been linked to a reduction in the number of service providers able to bid for treatment contracts [15, 38, 50]. The reduced budgets available to finance contracts is perceived as favouring non-National Health Service (NHS) to NHS providers [42]. It is also been linked to a reduction in the number of organisations applying for treatment contracts, excluding smaller local organisations and the evolution of treatment systems led by national organisations [15].

Meanwhile, the expertise of alcohol and drug treatment commissioners in England is under scrutiny [15, 39] with feedback from stakeholders that subject-specific expertise has been lost from commissioning teams as a result of staff turnover and an increase in the size and scope of commissioners’ portfolios [10, 15]. This is echoed in sexual health services which have been criticised as fragmented, with disjointed services and an increasing lack of accountability [50]. This includes examples of different aspects of services being commissioned via different bodies with diverse procurement approaches, resulting in disjointed pathways. This fragmentation in commissioning arrangements has also been criticised as leading to isolated disinvestment decisions, especially when cuts to one service have knock-on implications for other parts of the system.

A further contention within the local authority environment for public health is the fit with local political agendas [32, 36, 37, 42, 45, 47–49, 51]. Decisions about investment in a context of competing policy areas [49], investment choices being driven by popularity [38, 51], and not being able to align the benefits of public health services with local authority strategy [48] all appear to factor. Such differences across local authorities have been described as contributing to large variations in the prioritisation of public health agendas, investment and service provision [36, 48, 51].

### Health, social and other broader negative implications

Disinvestment from public health services has led to concerns about a downstream rise in demand on other publicly-funded services, and increases in communicable disease and crime [15, 32, 36, 39–41, 43, 45–47]. Editorials have highlighted that concurrent to disinvestment from other public health services, there have been deteriorating related outcomes, including increased rates of sexually transmitted diseases and teenage pregnancies, and a stagnation of the narrowing of socioeconomic gaps in life expectancy and quality of life [42, 48, 49, 51].

One English study, analysing routinely-collected secondary data, expressed concern about such disproportionate cuts to public health services contributing to widening health inequalities, with large variation in the quantity and quality of services available [46]. In a historical health impact study in the US [36], poorer health outcomes for low-income women were attributed to 30% cuts to family planning services .

Simultaneous to disinvestment from the alcohol and drug treatment sector have been increases in alcohol related hospital admissions and drug related deaths [15, 43, 45–47]. A historical health impact study in the US identified that policy decisions and budget cuts to public health services led to reduced availability of drug treatment [32]. The exponential rise in tuberculosis and HIV within the injecting drug treatment population - although the relationship was not formally analysed or modelled – was attributed to these budget cuts. Similar concerns have been raised in England more recently concerning the increasing number of drug-related deaths relating to fentanyl and how they might be linked to reduced needle exchange provision and associated support [44].

Furthermore, disinvestment appears linked to the withdrawal, or dilution, of services that support vulnerable groups [10, 40, 43]. For example, large disinvestment from substance use prison services has been linked to a lack of supported transition to community treatment, poor case management and a lack of Naloxone, potentially contributing to the rise in drug-related deaths [10, 40]. Similarly, people who may have previously benefited from targeted programmes [50] appear further marginalised following policy changes, including people in ethnic minority groups [10], people experiencing mental ill health and those with housing needs [10, 43].

## Discussion

The understanding of the impact of disinvestment is limited and no previous study has systematically examined the evidence. This study synthesises heterogeneous papers that provide insight as to how disinvestment from public health services might affect service provision and outcomes. Twenty papers were identified that contribute to understanding the impact of disinvestment from alcohol and drug treatment, and related public health services, in England and elsewhere. The review identified similarities between the described effects of disinvestment from alcohol and drug treatment services with the effects of disinvestment from broader public health services. The broader papers provide some additional empirical evidence in support of this review’s identified themes, including for example, poorer outcomes [37] and the effects of political influence [35].

Policy makers are facing challenging public health investment decisions during a time of sustained public austerity. There are numerous reported changes to the way services have been commissioned which may have negatively influenced treatment quality. Whilst perhaps driven by a need for efficiencies, service integration may have limited the specialisms within workforces and disproportionately impacted the alcohol treatment population.

The literature highlights concerns about the reduced quantity and quality of alcohol and drug treatment in England, following cuts to services. This is echoed in literature from other OECD countries and literature on disinvestment from other, similar public health services. However, there is limited exploration as to whether certain changes, including for example the integration of alcohol and drug treatment services, were done to limit direct impact of budget reductions. This study also identifies some evidence that disinvestment might be impacting more on some of the most disadvantaged areas, and vulnerable communities, potentially contributing to increasing health inequalities. Certain aspects of the treatment system are reported to have been disproportionately affected by budget cuts. Fewer harm reduction services and residential rehabilitation facilities, and less one on one time, may present particular challenges for people with more complex needs [21].

The influence of political agendas and competing pressures - where investment decisions are devolved - may be contributing to inconsistent investment and treatment provision. Disinvestment was often described in relation to the context of public sector austerity [15, 35, 36, 43, 46, 47] and how some cuts have been disproportionate to need [10, 38, 46, 52]. An English study highlighted an 8% reduction in expenditure on substance use services versus a 5% reduction in the available public health grant between 2013/14 and 2017/18 [38]. Two studies and an opinion piece also highlighted that local changes in investment in public health services in England had varied substantially between local authorities [39, 46, 51]. Some of the areas that had experienced the highest levels of alcohol and drug-related harm had reported some of the biggest percentage cuts to service budgets [43, 45, 46]. Investment decisions have been reported as being guided by political priorities and even personal stigmatisation of treatment populations [10, 32, 35, 37, 38, 41]. Given these concerns, and evidence that some vulnerable people may be being disproportionately affected by changes to treatment provision, it may be that disinvestment is contributing to widening health inequalities [53, 54].

Further to the themes identified in this review regarding the impact of disinvestment, there were substantial references within the literature to the context and conditions of disinvestment. Previous increases in investment were reported to have enabled innovation, for example, increased psychosocial support for people with alcohol and drug dependence and embedded support services within community settings [50]. Despite a reported substantial rise in investment in alcohol treatment between 2013/14 and 2015/16 [15], some claims were made within the literature that funding for alcohol has always been insufficient, with over two thirds of amalgamated budget being spent on drug treatment [15, 39, 43, 45].

Furthermore, the funding mechanisms devised to help protect public health grant funding in England (such as ring-fencing, to prevent expenditure on non-public health services) appear to have been limited in their success [15, 35, 38, 47, 48, 50]. These UK papers report public health grant funding being utilised to subsidise other local authority service provision, such as domestic abuse services, that do not fall within current *statutory* public health responsibilities. Within a context of local authority austerity, six papers highlighted stakeholder concerns that pressures on public health spending in the UK would further increase [15, 38, 41, 46, 47, 51], due to an expected decreasing public health grant and the intended removal of the ring-fence.

### Limitations of the study

The heterogeneity of the papers, in terms of the research methods employed and the way in which information was analysed and presented, limited our ability to synthesise results or make comparisons, leading us to choose a narrative-interpretive approach. The focus of this review and synthesis of diverse literature means that some of the results from individual papers will not have been detailed. The alcohol and drug treatment papers often failed to clearly outline the objectives or proposed analyses of their studies and therefore lacked transparency as to the measured outcomes or the criteria used to assess impact. This made it difficult to differentiate impacts associated with disinvestment from impacts associated with simultaneous commissioning, service provision and policy changes, or indeed the drivers of those changes. Whilst the literature about England clearly reports financial disinvestment from alcohol and drug treatment services and the perceived impact of these cuts, the association between the two and the accuracy of the published financial information, have not been studied. Furthermore, the drivers of disinvestment remain unclear, and how cuts have impacted on different elements of the treatment system, for example, different treatment modalities, or the configuration of services.

### Future research

This review has identified concepts which further empirical research should seek to examine to further advance the evidence of the impact of disinvestment from alcohol and drug treatment services, and other public health services. In England, for example, there are substantial routine data available to quantitatively examine the effects of disinvestment on treatment access and outcomes, as well as additional broader health harms. In countries where such data is available, it could be matched on a local geography or where available, matching patient and treatment data. This could help us to better understand variation in disinvestment and relative changes in treatment availability and effectiveness. As the systems that enable treatment appear complex and vary substantially, qualitative methods with key stakeholders could identify additional factors contributing to the effect of disinvestment. Within the reviewed literature, there is limited reference to attempts to moderate the impact of disinvestment and yet there are references to innovation in commissioning practices and service delivery during a period of sustained cuts. Further exploration of these factors may be helpful to support future decision-making to maintain treatment engagement and quality.

The important contextual factors to (dis)investment, regularly referenced within the literature, could be considered in future studies. For example, examining regional or socioeconomic variation in (dis)investment and treatment provision would help further advance our understanding as to whether budget cuts may be disproportionately affecting people living in deprived areas. Furthermore, research which seeks to understand local drivers of (dis)investment in alcohol and drug treatment services may also help to identify protective factors.

The quality appraisal of included research studies the literature highlighted some weaknesses in terms of study design and transparency in reporting. Therefore, future research should seek to fully report methods and use a quality checklist to improve its robustness.

## Conclusions

This study is the first to synthesise literature that explores the impact of disinvestment on alcohol and drug treatment and outcomes and identifies opportunities to further advance the body of evidence. In England, disinvestment from alcohol and drug treatment services has occurred in parallel to reduced public sector funding, declines in treatment outcomes and increases in alcohol-related hospital admissions and alcohol and drug-related deaths. However, the quantitative relationship between disinvestment from alcohol and drug treatment and related outcomes remains unexamined. Since the Health and Social Care Act 2012, substantial changes to the way in which services are commissioned and provided were reported. There was evidence of large variation in disinvestment across England with concerns about the potential for widening health inequalities. Given the known link between effective alcohol and drug treatment and reduced health and social harms, understanding the impact of disinvestment remains important to policy makers internationally. This may be particularly important given that disinvestment might result in increased pressure on more costly publicly funded services.

## Supplementary Information

**Additional file 1.**

## Data Availability

Not applicable.
