# Supplementary material for: The impact of disinvestment on alcohol and drug treatment delivery and outcomes: a systematic review
Source: BMC Public Health. 2021 Nov 22;21:2140. doi: 10.1186/s12889-021-12219-0 (PMC8609850; doi:10.1186/s12889-021-12219-0)
Supplement: Supplementary file 1 — Additional file 1. [file 12889_2021_12219_MOESM1_ESM.docx]

**Supplementary information**

Quality appraisals of reviewed literature

| ***CASP^a^ Systematic Review Quality Appraisal Tool: Literature reviews*** | | | | | | | | | | |
| --- | --- | --- | --- | --- | --- | --- | --- | --- | --- | --- |
| **Paper and year published** | **Q1**  Did the review address a clearly focused question? | **Q2**  Did the authors look for the right type of papers? | **Q3**  Do you think all the important, relevant studies were included? | **Q4**  Did the review's authors do enough to assess quality of the included studies? | **Q5**  If the results of the review have been combined, was it reasonable to do so? | **Q6**  What are the overall results of the review? | **Q7**  How precise are the results? | **Q8**  Can the results be applied to the local population? | **Q9**  Were all important outcomes considered? | **Q10**  Are the benefits worth the harms and costs? |
| Freudenberg et al. 2006 | No | Can't tell | Can't tell | No | Can't tell | The impact of a fiscal crisis and the resulting cots of a subsequent syndemic of Tuberculosis, HIV and homicide | Estimates | No | Yes | N/A |
| McFarlane 1993 | No | Yes | Can't tell | No | Yes | The negative impact of Reagan policies on public health services | Estimates | No | Yes | No |
| ***^a^*** *Critical Appraisal Skills Programme* | | | | | | | | | | |

| ***JBI^a^ Cross-Sectional Quality Appraisal Tool: Primary research papers*** | | | | | | | | |
| --- | --- | --- | --- | --- | --- | --- | --- | --- |
| **Paper and year published** | **Q1**  Were the criteria for inclusion in the sample clearly defined? | **Q2**  Were the study subjects and the setting described in detail? | **Q3**  Was the exposure measured in a valid and reliable way? | **Q4**  Were objective, standard criteria used for the measurement of the condition? | **Q5**  Were confounding factors identified? | **Q6**  Were strategies to deal with confounding factors stated? | **Q7**  Were the outcomes measured in a valid and reliable way? | **Q8**  Was appropriate statistical analysis used? |
| Anderson et al. 2017 | Yes | Yes | Yes | No | N/A | N/A | Yes | Yes |
| Chang et al. 2010 | Yes | Yes | Yes | Unclear | No | N/A | Yes | Yes |
| ***^a^*** *Joanne Briggs Institute* | | | | | | | | |

| ***JBI^a^ Text and Opinion Pieces Quality Appraisal Tool – journal published opinion pieces*** | | | | | | | |
| --- | --- | --- | --- | --- | --- | --- | --- |
| **Paper and year published** | **Q1**  Is the source of the opinion clearly identified? | **Q2**  Does the source of opinion have standing in the field of expertise? | **Q3**  Are the interests of the relevant population the central focus of the opinion? | **Q4**  Is the stated position the result of an analytical process, and is there logic in the opinion expressed? | **Q5**  Is there reference to the extant literature? | **Q6**  Is any incongruence with the literature/sources logically defended? | **Q7**  Is the opinion supported by peers? |
| Daube | Yes | Yes | Yes | Yes | No | No | Unclear |
|  |  |  |  |  |  |  |  |
| Iacobucci 2016 | Yes | No | Yes | No | No | No | Yes |
| Iacobucci 2014 | Yes | No | Yes | No | No | No | Yes |
| Mohammadi 2016 | Yes | No | Yes | Yes | No | No | Yes |
| White 2015 | Yes | No | Yes | No | No | No | Yes |
| ***^a^*** *Joanne Briggs Institute* | | | | | | | |

| ***AACODS^a^ Checklist for grey literature*** | | | | | | |
| --- | --- | --- | --- | --- | --- | --- |
| **Paper and year published** | **Authority** | **Accuracy** | **Coverage^b^** | **Objectivity** | **Date** | **Significance** |
| Adfam 2017 | Yes | Yes | ? | Yes | Yes | Yes |
| Advisory Council on the Misuse of Drugs 2017 | Yes | Yes | Yes | Yes | Yes | Yes |
| Alcohol Concern 2013 | Yes | Yes | No | Yes | Yes | Yes |
| Alcohol Concern 2018 | Yes | Yes | No | ? | Yes | Yes |
| Blenheim | Yes | No | No | ? | Yes | ? |
| British Medical Association 2018 | Yes | No | No | ? | Yes | Yes |
| Cook 2017 | Yes | Yes | Yes | Yes | Yes | Yes |
| Davies et al. 2016 | Yes | Yes | Yes | Yes | Yes | Yes |
| Drink and Drug News 2018 | ? | No | No | No | Yes | No |
| Robertson et al., 2017 | Yes | Yes | Yes | Yes | Yes | Yes |
| ***^a^*** *Authority Accuracy Coverage Objectivity Date Significance*  *^b^ This refers to whether the literature explicitly states its focus (population, questions) and therefore acknowledges its limitation* | | | | | | |
